# Community-level characteristics and environmental factors of child respiratory illnesses in Southern Arizona

**DOI:** 10.1186/s12889-017-4424-3

**Published:** 2017-05-25

**Authors:** Nathan Lothrop, Khaleel Hussaini, Dean Billheimer, Paloma Beamer

**Affiliations:** 1 Mel and Enid Zuckerman College of Public Health, University of Arizona, 1295 N. Martin Ave., PO 245210, Tucson, AZ 85724 USA; 2 Biomedical Informatics, College of Medicine, University of Arizona, Tucson, AZ 85724 USA; 3 BIO5 Institute, University of Arizona, Tucson, AZ 85724 USA; 4 Arizona Respiratory Center, University of Arizona, Tucson, AZ 85724 USA

**Keywords:** Asthma, Lower respiratory illness, Children, Neighborhood, Socio-economic status, Housing, Air pollution

## Abstract

**Background:**

Lower respiratory illnesses (LRIs) and asthma are common diseases in children <5 years of age. Few studies have investigated the relationships between multiple, home-based social and environmental risk factors and asthma and LRIs in children. Of those that have, none have focused exclusively on children <5 years of age, who are more physiologically vulnerable and spend more time at home compared to older children. Further, no studies have done so at the community level.

**Methods:**

We modeled relationships between emergency department visits and hospitalization rates for asthma and LRIs for children <5 years and geographic risk factors, including socio-economic and housing characteristics, ambient air pollution levels, and population density in Maricopa and Pima Counties, Arizona, from 2005 to 2009. We used a generalized linear model with a negative binomial observation distribution and an offset for the population of very young children in each tract. To reduce multicollinearity among predictors, socio-economic characteristics, and ambient air pollutant levels were combined into unit-less indices using the principal components analysis (PCA). Housing characteristics variables did not exhibit moderate-to-high correlations and thus were not included in PCA. Spatial autocorrelation among regression model residuals was assessed with the Global Moran’s I test.

**Results:**

Following the regression analyses, almost all predictors were significantly related to at least one disease outcome. Lower socio-economic status (SES) and reduced population density were associated with asthma hospitalization rates and both LRI outcomes (*p* values <0.001). After adjusting for differences between counties, Pima County residence was associated with lower asthma and LRI hospitalization rates. No spatial autocorrelation was found among multiple regression model residuals (*p* values >0.05).

**Conclusions:**

Our study revealed complex, multi-factorial associations between predictors and outcomes. Findings indicate that many rural areas with lower SES have distinct factors for childhood respiratory diseases that require further investigation. County-wide differences in maternal characteristics or agricultural land uses (not tested here) may also play a role in Pima County residence protecting against hospitalizations, when compared to Maricopa County. By better understanding this and other relationships, more focused public health interventions at the community level could be developed to reduce and better control these diseases in children <5 years, who are more physiologically vulnerable.

**Electronic supplementary material:**

The online version of this article (doi:10.1186/s12889-017-4424-3) contains supplementary material, which is available to authorized users.

## Background

Lower respiratory illnesses (LRIs) and associated complications are the leading cause of death in very young children [1], and LRIs and asthma are associated with increased morbidity [2]. In addition, asthma is among the most common childhood diseases in the United States (US), with 1,406,000 children under the age of 5 years diagnosed with asthma [3]. This is particularly important because the lungs of very young children are still developing, making them more susceptible to respiratory health risks such as air pollution, compared to adults [4, 5]. Additionally, very young children who have LRIs are more likely to develop respiratory issues later in life [6–8].

Asthma and LRI morbidity are influenced by complex relationships among social and environmental factors such as socio-economic status (SES), housing conditions, and ambient air pollution, in addition to genetic predispositions, lifestyle habits, and psychological stressors [9–13]. Past studies have linked children’s asthma and LRI emergency department (ED) visits and hospital admissions to a substantial list of social and environmental factors, including but not limited to lower household income, minority race [14], older housing structures [15], household crowding [16], and increased ambient air pollution [17–20]. Further, relationships among these factors and respiratory diseases have been found to vary across regions and spatial scales (e.g., census tract, county, state, etc.) [21–23].

While independent social and environmental risk factors have been identified for these diseases, few studies have examined multiple risk factors in children at the community level [12, 14, 15, 24]. Of those studies that have, none have focused exclusively on children <5 years of age, who are more physiologically vulnerable and spend more time at home (66% – 77% on average) compared to older children [25–27]. Combined with the impact these diseases in early childhood may have on respiratory health later in life [6–8], it is crucial to better understanding these relationships. By better understanding the complex, multi-factorial relationships between community-level predictors and these respiratory disease outcomes, we can better design public health interventions for specific areas or communities to reduce their prevalence in children under 5 years [28]. In this study, our goal was to better understand how socio-economic and housing characteristics, ambient air pollution levels, and geographic variables related to asthma and LRI ED visits and hospitalization rates in children under 5 years at a community level for years 2005–2009 in Maricopa and Pima Counties in Southern Arizona.

## Methods

### Study area and unit of analysis

**Fig. 1:**
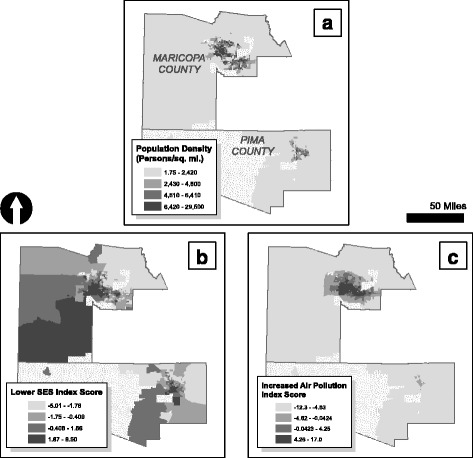
(**a**) Population density for areas included in analysis; (**b**) lower socio-economic status (SES) index score; and (**c**) increased air pollution index score. Note: stippled areas indicate tracts excluded from analyses, and data in maps are divided into quartiles

The study area includes Maricopa and Pima Counties, Arizona, United States (see Fig. 1a), which together account for 4,797,620 people (75% of the state’s total population of 6,634,997) [29]. Maricopa and Pima Counties have populations of 3,817,357 and 980,263 persons and average population densities of 415 and 107 persons per square mile, respectively [29]. The census tract is the smallest areal unit for which all data is available in the study area. Census tracts are geographic subdivisions of counties, which contain approximately 4000 people per tract, and have boundaries that are relatively stable through time. Together, these counties contain a total of 861 tracts (664 in Maricopa, 197 in Pima), however, only 826 tracts (96%) (636 in Maricopa, 190 in Pima) were included in the analyses. Tracts were excluded if: 1) they had incomplete hospital record data; 2) they had no children; or 3) they fell completely within tribal lands (i.e., on a Native American nation), as there are no statutory requirements for facilities on a Native American nation to report hospital discharge data.

### Outcome variables: rates of emergency discharge visits and hospitalizations for asthma and lower respiratory illnesses

The outcomes of this study were rates of ED visits and inpatient hospitalizations (hereafter referred to as “hospitalizations”) for primary diagnoses of asthma or an LRI (i.e., bronchitis, bronchiolitis, croup, or pneumonia) for children under 5 years aggregated by census tract for study years of 2005–2009. This study time period was chosen because Arizona’s population decreased noticeably in 2010 due to controversial state legislations regarding immigration. Population count and ethnicity data after 2010 was deemed too unreliable. Diagnosis count data aggregated at census tract level based on the 2000 US Census were obtained from the Arizona Department of Health Services (ADHS) for the study time period. For asthma, the Agency for Health Related Quality diagnosis code was used (*International Classification of Diseases* [ICD-10] code J45.*) [30]. For LRIs, including bronchitis, bronchiolitis, croup, and pneumonia, we used ICD-10 codes J40, J21.*, J05.0, and J18.9, respectively. The ‘*’ indicates that any number may be substituted. Only the primary diagnosis was used for this analysis.

In essence, a hospitalization occurs when a person is admitted to and later discharged from the hospital; an ED visit is when a person is admitted to the emergency department, not hospitalized, and later discharged, typically within 24 hours [31]. The unit of analysis for ED visit is the ED encounter, meaning a person who is seen in the ED multiple times in one year will be counted each time as a separate ED “encounter” [32]. While ED visits and hospitalization rates are both sensitive to health care access, ED visit rates are considered a better marker of the lack of disease controlling medications or regular healthcare visits, compared to hospitalizations, which better represent disease severity [33, 34]. While hospitalizations and ED visit rates are not synonymous with disease prevalence, these data allow us to study relationships between geographic factors and disease outcomes over a large geographic area (2 US counties).

### Explanatory variables: geographic factors

**Fig. 2:**
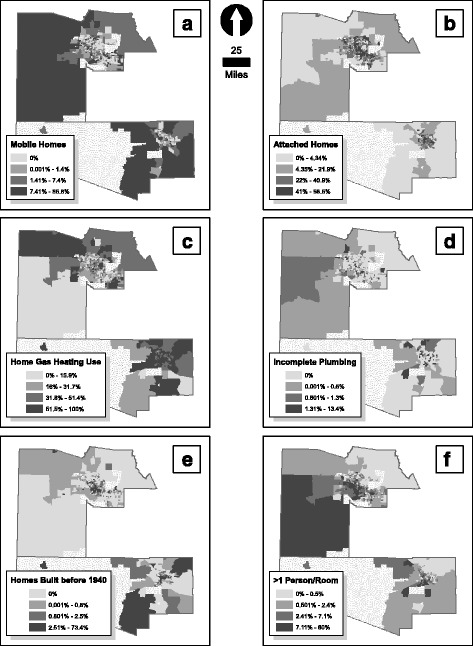
Proportion of **a** mobile homes; **b** attached homes; **c** home gas heating use; **d** homes with incomplete plumbing; **e** homes built before 1940; and **f** homes with >1 person/room. Note: stippled areas in maps indicate tracts excluded from analyses, and data in maps are divided into quartiles

Explanatory variables were extracted from previous studies that have investigated social and environmental factors related to asthma and LRIs in young children [14–16, 35]. Factors were separated into categories of SES, housing characteristics, and ambient air pollution. Population density and county (i.e., Maricopa and Pima) were also analyzed but not included in any of the categories. Maps were created of population density and housing characteristics (Figs. 1a and 2) with factor values divided into quartiles. We obtained SES and housing variables from the 2005–2009 US Census American Community Survey [36]. Variables included in the SES category were not related to the physical environment (i.e., housing, air pollution levels) or geography (i.e., population density, county), and generally included age, country of birth, gender, race, language spoken at home, per capita income, employment status, and education. Additional descriptions of variables in SES and ambient air pollution categories are available in Additional file 1: Table S1.

Modeled ambient air pollution concentrations were attained from US Environmental Protection Agency’s 2005 National Air Toxics Assessment (NATA) [37]. NATA was developed to estimate annual air pollution levels at the census tract-level for the entire US, which lacks spatially-representative air pollution monitoring networks [38]. The 2005 NATA dataset is the result of federal, state, and local agencies inventorying outdoor stationary and mobile sources of air pollution, and then estimating ambient annual average concentrations of air pollutants using air dispersion and photochemical models [39]. In our study area, major sources of pollution in NATA data include point and non-point sources. Major point source emissions from both counties include a mix of industrial factories and landfill operations. Maricopa County has a more extensive network of freeways and rail lines compared to Pima County. In relation to other areas in the US, both counties are below the annual average levels of particulate matter size < 2.5 μm in diameter, a product of fuel combustion. Each individual variable was log-transformed to reduce skew and kurtosis, and was then standardized.

We analyzed the correlation among variables adjusting for multiple comparisons by category (i.e., SES, housing, air pollution) using a Bonferroni corrected Pearson correlation, which reduces the risk of finding false correlations by chance (i.e., a Type I Error) among three or more variables [40]. If variables exhibited moderate-to-high correlations (i.e., ρ > 0.30 and *p* < 0.05) among those in their respective category (i.e., SES, housing, air pollution), variables in that category were combined into a unitless index using principal components analysis (PCA). PCA is a technique for reducing large data sets, where the loading of each variable illustrates the correlation between that variable and the component [41]. In this process, linear relationships among variables are found (components), with each component being uncorrelated with the others (orthogonal) in directions defined by an eigenvector. The variances extracted by the components are called the eigenvalues. The most ‘principal’ components account for most of the total variance among variables. We considered the inverse of median age and per capita income to ensure factors were positively correlated. Thus, if variables were combined by category using PCA, the result would be an index of a category representing variables of said category. For example, SES variables that denoted lower or poorer SES (e.g., lower per capita income) were combined using PCA, yielding a first principal component score of “lower SES”. Therefore, the higher the score of “lower SES”, the lower the SES; and vice versa. In all cases, the first principal component score was used and standardized prior to regression analyses. We mapped the first principal component scores for SES and air pollutants as indices of lower SES and increased air pollution (Fig. 1b, c), with scoring divided into quartiles. We also determined differences in factors by county using the two-sample t-test. Correlations, PCA, and t-tests were computed in STATA 13.0 (StataCorp, College Station, TX). An α < 0.05 was used to determine the statistical significance. All maps were created in ArcMap 10.1 (ESRI, Redlands, CA).

### Statistical approach

We used a generalized linear model with a negative binomial observation distribution and an offset for the log-transformed population of children <5 years of age for each tract to model ED visits and hospitalization rates for asthma and LRIs. The negative binomial model extends the simpler Poisson regression model by allowing over-dispersion of the disease counts [42]. Initially, we tested our models using zero-inflated negative binomial models; however, the regular negative binomial produced lower Bayesian Information Criterion scores, indicating a better model fit [42]. After this initial testing, we pre-determined the variables of interest and did not do the model selection so we could better understand the complex relationships and the relative importance of predictors in disease outcomes. We judged the relative importance of each factor based on the magnitude of the incidence rate ratio (IRR). The IRR represents the change in the outcome in terms of a percent increase or decrease, with the percentage determined by the amount the IRR is above or below 1 (i.e., no change). The 95% Confidence Interval (95% CI) indicates that there is a 95% probability that the true IRR will lie in the range of the 95% CI, assuming no biases or confounding. Both simple and multiple negative binomial regressions were completed using SAS 9.3 (SAS Institute, Cary, NC), and α < 0.05 was used to determine the statistical significance. We used a Global Moran’s I test in GeoDa Version 1.6.5 (GeoDa Center, Tempe, AZ) to test for spatial autocorrelation among multiple regression model residuals. Spatial autocorrelation refers to the tendency for a variable to be correlated with itself through geographic space. Spatial autocorrelation exhibited among the residuals will not satisfy the assumption of independence among observations [43].

## Results

### Emergency discharge visits and hospitalization counts for asthma and lower respiratory illnesses

All 826 tracts included in analyses had ED visits data, while only 805 tracts had hospitalization data. The 21 tracts (2.5% of 826 tracts) without hospitalization count data were all in Pima County. Statistics for ED visit and hospitalization counts can be found in Table 1. Overall, there were more diagnoses for LRIs compared to asthma for both ED visits and hospitalization data. For asthma, most tracts in the study area experienced five or fewer ED visit or hospitalization events during the study period. The maximum number of events were 153 and 250 for ED visits and hospitalizations, respectively. For LRIs, most tracts experienced approximately 51 ED visits, with a maximum of 943 visits. The maximum value for LRI hospitalization counts was 117, while more than half of tracts experienced eight or more hospitalizations during the study period.

**Table 1:** Primary disease ED visits and hospitalization counts by census tract

Disease outcome	Record type	Number	Minimum	Median	Maximum	Skewness	Kurtosis
Asthma	ED visits	826	0	4.50	153	5.52	53.9
	Hospitalizations	805	0	0	250	16.5	377
LRIs	ED visits	826	0	51	943	4.14	26.4
	Hospitalizations	805	0	8	117	3.45	19.8

### Assessment of geographic factors

Using PCA, we reduced the SES and air pollution variable into unitless indices in their respective categories. Ten socio-economic variables of interest and 47 air pollutants’ concentrations were combined to form indices of ‘lower SES’ and ‘increased air pollution,’ respectively. Of the 178 pollutants available in the 2005 NATA data, 131 were excluded because they exhibited limited spatial variability within the study area. Pollutants associated with increased respiratory diseases, such as ozone [44], are primarily associated with mobile emissions [45] and are included in the 2005 NATA data. All socio-economic characteristics and air pollution concentration variables were positively correlated with their respective first principal component, which represented 56% and 72% of the variance, respectively. Thus, higher scores of the lower SES index show areas that are more socio-economically distressed, and higher scores of the increased air pollution index illustrate areas that have an increased burden of ambient air pollution based on 2005 NATA data. Maps of the indices are found in Fig. 1b and c, and first principal component scoring coefficients of socio-economic characteristics and ambient air pollutant concentrations are shown in Additional file 1: Table S1.

Population density ranged from 1.75 to 29,500 persons/sq. mile (Table 2). Housing characteristics variables did not exhibit moderate-to-high correlation (i.e., ρ > 0.30 and *p* < 0.05) using a Bonferroni corrected Pearson correlation (see Additional file 1: Table S2). The first principal component accounted for only 28% of the variance in housing characteristics data. Therefore, these data were not combined into a unitless index using PCA. Housing characteristics exhibited a wide range of proportions by census tract (Table 2). For example, on average by census tract, 34% of homes used home gas heating (95% CI = 5.13%–6.99%), while 0.37% homes lacked complete plumbing (95% CI = 0.30%–0.44%). Maps of population density and housing characteristics are shown in Figs. 1a and 2, respectively.

**Table 2:** Comparison of study population and factor variables (*n* = 826) using a two-sample t-test

Variable	Maricopa County				Pima County			
	Range	Median	Mean	SD	Range	Median	Mean	SD
Study population	8–7105	364	507	611	7–1,930	262	352	306
Lower SES	−5.01 - 8.50	−0.35	0.04	2.44	−5.01 - 4.95	−0.54	−0.13	2.16
Increased air pollution***	−10.0 - 17.0	1.62	2.05	4.85	−12.3 - -0.75	−6.84	−6.88	2.52
Population density (persons/sq. mile)***	1.75–29,500	4900	5200	3300	3.08–9, 300	2920	3300	2400
Housing characteristics								
Mobile homes (%)***	0.00–86.8	0.60	4.86	11.1	0.00–76.8	1.40	10.1	19.2
Attached homes (%)*	0.00–98.6	22.5	26.9	24.0	0.00–80.7	19.7	22.3	20.6
Home gas heating (%)***	0.00–100	25.2	27.3	18.5	10.5–88.1	59.2	57.4	16.0
Lacking complete plumbing (%)	0.00–13.4	0.00	0.36	1.06	0.00–3.40	0.00	0.41	0.75
Built before 1940 (%)*	0.00–73.4	0.00	1.53	5.61	0.00–58.1	0.30	2.84	7.78
> 1 person per room (%)**	0.00–60.0	2.70	5.54	7.15	0.00–22.5	1.55	3.77	5.17

Note: *p < 0.05; **p < 0.01; ***p < 0.001

For all 826 analysis tracts, Maricopa County had significantly higher air pollution index scores (*p* < 0.001), proportions of attached homes (*p* = 0.02) and homes with >1 person/room (*p* < 0.001), and population densities (*p* = 0.002) (Table 2). Meanwhile, Pima County had significantly higher proportions of mobile homes (*p* < 0.001), home gas heating use (*p* < 0.001), and homes built before 1940 (*p* = 0.01). There was no significant difference between counties for SES index score (*p* = 0.38) or proportion of homes with incomplete plumbing (*p* = 0.50). These relations were consistent when only the 805 tracts used for hospitalizations were analyzed. The 21 tracts excluded from analyses from the hospitalization count outcomes had significantly higher SES (*p* < 0.001) and higher proportions of homes with gas heating (*p* < 0.001). The 805 tracts included in hospitalization count analyses had significantly higher air pollution index scores (*p* < 0.001), population densities (*p* < 0.001), and proportions of homes with household crowding (*p* = 0.003). There was no significant difference between these census tracts in the proportion of mobile homes (*p* = 0.99), attached homes (*p* = 0.14), homes with incomplete plumbing (*p* = 0.38), and homes built before 1940 (*p* = 0.67).

### Associations between factors and asthma and lower respiratory illness outcomes

In simple negative binomial regressions for primary diagnoses, nearly all variables were significantly associated with an outcome (Table 3). For asthma ED visit rates, the proportion of mobile homes and homes lacking complete plumbing were the only variables not significantly associated. For asthma hospitalization rates, the proportion of attached homes and homes built before 1940, county, and population density were unrelated. The proportion of homes with gas heating and county were not associated with LRI ED visit rates, while all variables were significantly associated with LRI hospitalization rates.

**Table 3:** Negative binomial regression analyses of asthma and LRI ED visit and hospitalization rates and factors

	Asthma		LRIs	
	ED Visits	Hospitalizations	ED Visits	Hospitalizations
Risk factors	IRR (95% CI)	IRR (95% CI)	IRR (95% CI)	IRR (95% CI)
Simple analyses				
Lower SES	1.35 (1.26–1.46)***	1.47 (1.30–1.67)***	1.11 (1.09–1.13)***	1.44 (1.33–1.56)***
Increased air pollution	1.33 (1.24–1.42)***	1.16 (1.03–1.31)*	1.03 (1.02–1.04)***	1.16 (1.08–1.25)***
Mobile homes (%)	1.03 (0.99–1.07)	1.10 (1.03–1.17)**	1.05 (1.03–1.07)***	1.10 (1.06–1.15)***
Attached homes (%)	1.09 (1.05–1.13)***	1.03 (0.97–1.10)	1.07 (1.05–1.09)***	1.05 (1.01–1.10)**
Home gas heating (%)	0.89 (0.83–0.95)***	1.16 (1.02–1.32)*	0.99 (0.96–1.03)	1.14 (1.05–1.23)**
Lacking complete plumbing (%)	1.06 (0.99–1.12)	1.16 (1.05–1.29)**	1.07 (1.03–1.10)***	1.20 (1.12–1.29)***
Built before 1940 (%)	1.12 (1.08–1.17)***	1.05 (0.98–1.14)	1.09 (1.06–1.11)***	1.11 (1.06–1.17)***
> 1 person per room (%)	1.21 (1.15–1.26)***	1.25 (1.16–1.34)***	1.12 (1.10–1.14)***	1.22 (1.17–1.28)***
Population density (persons/sq. mile)	1.20 (1.12–1.30)***	0.91 (0.80–1.04)	1.05 (1.01–1.08)*	0.80 (0.73–0.87)***
In Pima County *(Ref: Maricopa County)*	0.66 (0.54–0.80)***	0.83 (0.60–1.14)	0.92 (0.85–1.01)	0.80 (0.66–0.98)*
Multiple analysis				
Lower SES	1.15 (0.99–1.33)	1.57 (1.24–1.97)***	1.21 (1.13–1.28)***	1.52 (1.32–1.76)***
Increased air pollution	1.05 (0.93–1.19)	0.87 (0.72–1.05)	1.12 (1.06–1.19)***	0.90 (0.79–1.01)
Mobile homes (%)	1.01 (0.97–1.05)	1.03 (0.97–1.10)	1.03 (1.01–1.05)**	1.04 (1.01–1.08)*
Attached homes (%)	1.01 (0.96–1.05)	1.02 (0.95–1.09)	1.04 (1.02–1.06)***	1.04 (1.00–1.08)
Home gas heating (%)	0.91 (0.84–0.98)**	1.18 (1.03–1.35)*	0.98 (0.95–1.02)	1.11 (1.03–1.2)**
Lacking complete plumbing (%)	1.02 (0.96–1.08)	1.07 (0.97–1.19)	1.01 (0.98–1.04)	1.09 (1.02–1.16)*
Built before 1940 (%)	1.07 (1.02–1.13)**	0.93 (0.86–1.01)	1.02 (0.99–1.04)	0.97 (0.92–1.02)
> 1 person per room (%)	1.06 (0.97–1.15)	1.07 (0.94–1.22)	0.99 (0.96–1.02)	1.05 (0.97–1.13)
Population density (persons/sq. mile)	1.01 (0.88–1.15)	0.55 (0.45–0.67)***	0.94 (0.89–1.00)*	0.50 (0.45–0.57)***
In Pima County *(Ref: Maricopa County)*	0.77 (0.59–1.01)	0.47 (0.31–0.73)***	1.07 (0.95–1.21)	0.48 (0.38–0.62)***

Note: *CI* confidence interval, *IRR* incidence rate ratio; *p < 0.05; **p < 0.01; ***p < 0.001

In multiple regressions, asthma outcomes had fewer significant predictors compared to LRIs (two for ED visits and four for hospitalizations vs. five and six, respectively) (Table 3). Lower SES and reduced population density were significantly related to all outcomes except asthma ED visit rates. Home gas heating use was protective against asthma ED visits but was related to hospitalizations for asthma and LRIs. Living in Pima County protected against hospitalizations. The proportion of mobile homes was a factor for LRI outcomes.

Following the multiple regression analyses, the tracts with increased asthma ED visit rates would be expected to have a lower proportion of homes with gas heating and a larger proportion of homes built before 1940. Meanwhile, asthma hospitalization rates were associated with lower SES and the proportion of homes using gas heating, living in Maricopa County, and reduced population density. LRI ED visit rates were related to lower SES, increased air pollution levels, proportions of mobile homes and attached homes, and reduced population density. Meanwhile, LRI hospitalization rates were associated with lower SES, reduced population density, living in Maricopa County, and proportions of mobile homes, gas heating use, and homes with incomplete plumbing. Household crowding was not a significantly related to any outcome. Model residuals did not exhibit significant spatial autocorrelation. Simple and multiple regressions predicting the same outcomes for any diagnosis LRIs were nearly identical to primary diagnosis LRIs and can be found in Additional file 1: Table S3.

## Discussion

While independent social and environmental risk factors have been identified for these diseases, few studies have examined their relationship to multiple predictors at the neighborhood level [12, 14, 15, 24, 46]. Of those studies that have, none have focused exclusively on children <5 years, who are more physiologically vulnerable and spend more of their time at home (ranging from 66 to 77% on average) compared to older children [25–27]. In our study, we investigated the relationships between socio-economic and housing characteristics, ambient air pollution levels, and geographic variables and asthma and LRI ED visits and hospitalization rates in children under 5 years at a community level in Maricopa and Pima Counties in Southern Arizona. Socio-economic characteristics and ambient air pollutant levels were combined into unitless indices (i.e., lower SES and increased air pollution levels) using PCA, accounting for 56% and 72% of variance, respectively. Housing characteristics variables did not exhibit moderate-to-high correlations (i.e., ρ > 0.30 and *p* < 0.05 in a Bonferroni-corrected Pearson correlation) and thus were not combined using PCA. Nearly all predictors, except household crowding, were significantly related to one or more outcomes in the multiple regressions. Notably, lower SES and reduced population density were associated with asthma hospitalization and both LRI outcomes. Living in Pima County was protective against asthma and LRI hospitalization, when compared to Maricopa County, which had significantly higher air pollution levels, among other factors (Table 2). Multiple regression model residuals did not exhibit spatial autocorrelation, satisfying the regression assumption of independent observations. Our results have provided more information on the complex, multi-factorial relationships between asthma and LRIs and geographic factors at the neighborhood level based off home census tract. By better understanding these relationships, it may be possible to design more focused public health interventions at the community level to prevent and better control these diseases in children under 5 years, who spend most of their time at home and are more physiologically vulnerable compared to older children.

When predicting ED visits and hospitalization rates for asthma and LRIs, we found lower SES was significantly related to all outcomes except for asthma ED visit rates, which bordered on significance (Table 3). Other studies have associated asthma hospitalizations with various measures of lower SES, including minority race, household income, unemployment, adult education levels, and proportion of non-English language speaking persons [14, 15, 47, 48]. We also found lower SES was associated with LRI hospitalization rates, as has been shown previously [24, 49, 50]. Both findings suggest a lack of financial resources to obtain regular medical care or controlling medications, thus waiting till symptoms become so severe that the child will be hospitalized [15, 51]. Another potential contributor to these relationships could be the association of parental and household smoking and increased risk of LRIs [52]. Although tract-level data for smoking rates were not available for this study, it is known that lower SES [53] and education levels are associated with smoking rates [54]. In addition, minorities or those with limited English language skills may receive substandard care during and after hospitalization, potentially leading to repeat hospitalizations [55]. Future analyses accounting for hospital readmissions may better elucidate these complex relationships. Nevertheless, our findings indicate that very young children living in census tracts with fewer financial or health resources to control asthma or LRIs are more likely to be hospitalized for these diseases.

Reduced population density was significantly related to all outcomes but asthma ED visit rates, suggesting that rural areas may have more severe cases of respiratory diseases that may result from parents waiting until the symptoms become so extreme that a hospital visit is necessitated. Pesek et al. [56] found that children living in rural Arkansas have more undiagnosed and severe asthma symptoms compared to children in urban areas after accounting for other factors [56]. In another study of very young children from a Medicaid cohort in Tennessee, rural participants were more likely to have asthma and visit the ED for asthma-related incidents and were less likely to use asthma medications (inhaled corticosteroids), compared to their urban counterparts [57]. Valet et al. also found that rural children were more likely to have mothers who smoked [57], which is associated with more severe respiratory symptoms [58]. This contradicts other studies which found children in rural non-farm areas were less likely to develop asthma than those in urban settings [59] or were no different than those in urban areas [60]. However, these studies were not conducted with older children, whose lung function and development of asthma were likely already determined by unknown early life exposures [28, 61]. Our findings suggest that urban areas have more health resources to control asthma and LRIs (e.g., more transportation options and quicker ambulance response times [62]) before they become so severe they require hospitalization. Future studies should incorporate more information about these potential explanatory variables such as household transportation options, insurance status, and distance and effort to access regular health care to better understand the complex relationships between population density and respiratory diseases. Moving forward, telemedicine as a means to access care providers and specialists, has shown to be an effective means of reaching respiratory specialists [63] and reducing asthma intensity in underserved communities [64]. Another potential solution may be coordinating care among clinics, child care facilities, and caregivers to improve asthma outcomes [65].

In addition to population density, county of residence was also related to respiratory disease outcomes. We found that living in Pima County was negatively associated with asthma and LRI hospitalization rates, even after accounting for differences in predictors by county (Table 2). Maricopa County had significantly more air pollution (Interquartile range [IQR] = −0.97–5.39, Coefficient of Variation [CV] = 236) and greater proportions of attached homes (IQR = 1.93%–42.4%, CV = 89.3) and household crowding (IQR = 0.70%–7.70%, CV = 130) and population density (IQR = 2,900–6,770 persons/sq. mile, CV = 62.9), while Pima County had significantly larger proportions of mobile homes (IQR = 0%–9.4%, CV = 190), home gas heating use (IQR = 47.8%–69.1%, CV = 27.8), and homes built before 1940 (IQR = 0%–1.90%, CV = 274). Other explanations of Maricopa County’s increased respiratory disease rates could be explained by a number of factors, such as higher pregnancy rates among females 18–19 years of age during the study period [66]. Children of younger mothers are more likely to have wheezing LRIs than those born to older ones [67], and children born to younger mothers are more susceptible to environmental factors, such as diesel traffic related air pollution [68]. Another potential explanation could be that Maricopa County has ten times the proportion of land used for crop production compared to Pima County, which may lead to increased pesticide exposure in nearby residences, promoting childhood respiratory diseases [69]. Further, Maricopa County has more industrial livestock operations, which have been linked to childhood asthma and other respiratory issues [70, 71]. Another potential explanation could be that, in areas where employers are primarily agricultural, they may not offer insurance, leading to delays in seeking out care [72].

Interestingly, increased air pollution was significantly related to all outcomes in the simple regressions but was only related to LRI ED visit rates when accounting for other factors. Darrow et al. [73] also found this same relationship, specifically during abrupt increases in traffic-related air pollution due to meteorological changes. This could be a feasible explanation for findings in our study area, however one which we do not have the temporal resolution in air pollution data to address. In addition, because air pollution concentrations are based on emissions inventories, this may result in exposure misclassification, leading to underestimating the relationship of air pollution and respiratory disease outcomes. This relationship may increase in strength and significance, if exposure estimates have more spatial variability [74]. It is also important to note that allowable concentrations of criteria air pollutants governed by the US National Ambient Air Quality Standards were reduced after our study [75–78]. While this is beyond the scope of our project, it might further reduce air pollution’s significance as a predictor of respiratory disease when accounting for other factors. By accounting for lower levels of air pollutants in future studies, it might be possible to further elucidate these complex relationships among factors and outcomes.

For home characteristics, the proportion of home gas heating use was associated with asthma and LRI hospitalization rates, yet negatively associated with asthma ED visit rates. Other studies have found that exposure to gas combustion sources, whether for heating or cooking, have led to increased LRIs [79–81]. However, our study also showed that the proportion of gas heating use was negatively related to asthma ED visit rates, running contradictory to associations with hospitalization rates for asthma and LRIs. A similar but not significant relationship was found with LRI ED visit rates and home gas heating use (IRR = 0.98; 95% CI = 0.95–1.02). Meanwhile, hospitalization outcomes were significantly related to the proportion of home gas heating. These contrasting relationships between ED and hospitalization rates and proportion of gas heating may result from 826 tracts having ED outcome data, compared to just 805 tracts for hospitalization outcomes. The 21 tracts without hospitalization data have significantly increased SES and reduced air pollution levels and proportions of household crowding and population density compared to the other 805 tracts (Wilcoxon rank-sum test; *p* < 0.001 for all variables). This suggests that while home gas heating use relates to severity of respiratory diseases, it has a more nuanced relationship to care access in certain areas. This may be elucidated with future study into factors such as transportation options, insurance status, and availability of respiratory diseases specialists.

The proportions of mobile and attached homes were both associated with LRI ED visit rates. The relationship between LRI hospitalization rates and proportions of mobile homes may result from moisture build up from poorer ventilation [82], however there are no recent studies examining these relationships. This could also suggest that residents in areas with high proportions of mobile homes (more common in rural areas) wait until LRI symptoms are so severe that the children require hospitalization. Also, the proportion of mobile homes was significantly related to LRI hospitalizations, while attached homes had a similar but insignificant relationship (IRR = 1.03; 95% CI = 0.99–1.08). This may suggest that residents of areas with greater proportion of attached homes (more common in more urban areas) also wait until symptoms are severe, but simply because they are closer to care, they take less time to reach the ED, and as a result, have less severe symptoms compared to those traveling a further distance in rural areas. Our results are similar to findings of increased health disparities for residents in rural areas with lower population density (increased proportion of mobile homes) and in old urban cores of Phoenix and Tucson with higher population densities (increased proportion of attached homes) [83].

Similarly, homes built before 1940 were related to asthma ED visit rates, and the top 25% of tracts with high proportions of older homes were in rural areas and old urban cores of Phoenix and Tucson (Fig. 2). Again, this may indicate areas lacking care access either due to geographic proximity (rural areas) or lack of insurance (old urban cores). Questions of access may be answered in the future with more comprehensive GIS datasets of health care provider locations and transportation options. The proportion of homes with incomplete plumbing, likely an indicator of poor sanitation, was also related to LRI hospitalization rates. This same relationship between plumbing status and LRIs in young children has also been shown, albeit in a very different environment (i.e., Alaska) [84]. Interestingly, household overcrowding, which Luijk et al. [85] identified as a predictor for asthma and LRI symptoms, was related to all outcomes in the simple regressions but none in the multiple regressions. This may suggest that, while crowding is a potential factor, it is overtaken by others in our study area or may be indicative of other characteristics, such as parity [86] or bed-sharing [85].

Our study has several limitations, notably that this study was completed prior to the implementation of the Affordable Care Act, which may now alter relationships among geographic factors and outcomes due to a changed insurance landscape. In addition, the population in our study area grew from 2005 to 2009, which could increase the chances of model mis-specification. While asthma should not be diagnosed until after age 5 or 6 [87], because of the high number of children in the transient wheeze phenotype before age 6 years who do not go on to develop asthma [61, 88], we felt it important to assess factors for children <5 years because this may predict the expression of asthma and lung function in childhood and beyond [28, 61]. Instead, asthma diagnoses before age 5 or 6 may reflect measures of care quality and disease severity (ED and hospitalization visits, respectively) [89]. Despite these shortcomings, our paper has numerous strengths including the use of PCA to reduce many correlated predictor variables into unitless indices. This let us assess multiple known risk factors for childhood respiratory diseases in very young children for a large area (2 counties with 4.8 million residents). We also included spatial variables (population density and county), which helped to decrease the chance for residuals to exhibit spatial autocorrelation. As a result, our models meet the assumption of independent observations, while also accounting for natural spatial relationships among observations (i.e., census tracts) [90]. Furthermore, we studied respiratory diseases in children under 5 years, who are more susceptible to these factors yet not studied with these outcomes and predictors at the neighborhood scale.

## Conclusions

In our study, we found several geographic factors that are common predictors of both disease severity and access to care for asthma and LRIs at the neighborhood level. While other studies have investigated multiple risk factors for respiratory disease, we believe we are the first to focus on the physiologically vulnerable population of children <5 years of age during a critical time in their lung development. Our findings support other studies linking various social and environmental factors (e.g., lower SES) to asthma and LRI respiratory ED visits and hospitalizations. Our study also illustrates how (based on the magnitude of IRRs in multiple regressions) decreased population density and living in Maricopa County were the strongest predictors of hospitalizations (i.e., increased disease severity), even when controlling for other factors. These findings indicate that mostly rural areas with lower SES have distinct factors related to respiratory diseases in very young children that require further investigation. There are interventions used in older children in rural areas that could be adapted to our study age group (e.g., telemedicine and coordinated care among clinics, day cares, and caregivers). Differences between counties in factors not investigated here (e.g., maternal characteristics, agricultural land use) need more study. By incorporating these and other potential factors, future studies may further elucidate the complex, multi-factorial relationships between these factors and outcomes which we have brought to light. In doing so, public health interventions can be tailored to specific geographic areas at the community level to reduce the respiratory diseases burden on very young children, who are mostly vulnerable and whose future health can be greatly influenced by these diseases.

## Additional file

Additional file 1: Additional summary statistics of predictors and any and primary diagnosis for LRIs for ED visit and hospitalization rates. **Table ﻿S1.** Summary and first principal component scoring coefficient of socioeconomic characteristics and air pollution variables by census tract (*n* = 826). **Table S2.** Bonferroni-corrected Pearson correlation coefficient of housing characteristics. **Table S3.** Negative binomial regression analyses of any and primary diagnosis of LRIs for ED visit and hospitalization rates and factors. (DOCX 30 kb)

## Abbreviations

ADHS: Arizona Department of Health Services
CI: 95% confidence interval
CV: Coefficient of Variation
ED: Emergency department
ICD: International Classification of Diseases
IQR: Interquartile Range
IRR: Incidence rate ratio
LRI: Lower respiratory illness
NATA: National Air Toxics Assessment
PCA: Principal components analysis
SES: Socio-economic status
US: United States
