# Supplementary material for: Community-level characteristics and environmental factors of child respiratory illnesses in Southern Arizona
Source: BMC Public Health. 2017 May 25;17:516. doi: 10.1186/s12889-017-4424-3 (PMC5445507; doi:10.1186/s12889-017-4424-3)
Supplement: Additional file 1: — Additional summary statistics of predictors and any and primary diagnosis for LRIs for ED visit and hospitalization rates. Table ﻿S1. Summary and first principal component scoring coefficient of socioeconomic characteristics and air pollution variables by census tract (n = 826). Table S2. Bonferroni-corrected Pearson correlation coefficient of housing characteristics. Table S3. Negative binomial regression analyses of any and primary diagnosis of LRIs for ED visit and hospitalization rates and factors. (DOCX 30 kb) [file 12889_2017_4424_MOESM1_ESM.docx]

**Table S1.** Summary and first principal component scoring coefficient of socioeconomic characteristics and air pollution variables by census tract (n=826).

| **Variable** | **Mean** | **SD** | **Min.** | **Max.** | **Median** | **Score** |
| --- | --- | --- | --- | --- | --- | --- |
| ***Socioeconomic Characteristics*** |  |  |  |  |  |  |
| Median Age (years) | 35.7 | 9.28 | 12.5 | 76.6 | 34.1 | 0.33 |
| Foreign Born (%) | 16.3 | 11.5 | 0.00 | 61.5 | 12.0 | 0.34 |
| Male (%) | 50.3 | 4.43 | 33.7 | 78.9 | 49.9 | 0.18 |
| Minority Race (%) | 20.6 | 12.1 | 0.60 | 65.1 | 18.4 | 0.32 |
| Spanish Origin (%) | 31.4 | 25.2 | 0.80 | 97.0 | 21.3 | 0.39 |
| Per capita income ($) | 26,800 | 13,400 | 2,400 | 107,000 | 24,700 | 0.33 |
| Family with single female head of house (%) | 12.5 | 6.92 | 0.00 | 61.8 | 11.6 | 0.27 |
| Do not speak English at home (%) | 28.7 | 21.2 | 2.90 | 86.6 | 19.3 | 0.39 |
| Unemployed ≥ 15 weeks (%) | 27.1 | 9.26 | 8.50 | 83.4 | 25.6 | 0.21 |
| ≤12 years of school (%) | 8.92 | 7.20 | 0.00 | 59.8 | 7.00 | 0.34 |
| ***Ambient Air Pollutant Concentrations (µg/m^3^)*** |  |  |  |  |  |  |
| *Major Air Pollutants* |  |  |  |  |  |  |
| Diesel Particulate Matter | 1.69E+0 | 1.43E+0 | 2.59E-2 | 1.13E+1 | 1.44E+0 | 0.15 |
| *Polycyclic Aromatic Hydrocarbons* |  |  |  |  |  |  |
| Naphthalene | 6.83E-2 | 3.84E-2 | 5.31E-3 | 2.48E-1 | 6.66E-2 | 0.16 |
| PAHPOM (Polycyclic Organic Matter) | 1.12E-2 | 6.24E-3 | 1.52E-4 | 4.12E-2 | 1.05E-2 | 0.14 |
| *Organic Compounds* |  |  |  |  |  |  |
| Toluene | 3.32E+0 | 1.48E+0 | 9.10E-1 | 1.08E+1 | 3.16E+0 | 0.16 |
| Formaldehyde | 3.08E+0 | 7.63E-1 | 1.27E+0 | 5.78E+0 | 3.09E+0 | 0.15 |
| Acetaldehyde | 2.99E+0 | 4.83E-1 | 1.62E+0 | 4.18E+0 | 3.05E+0 | 0.15 |
| Xylenes (isomers and mixture) | 1.36E+0 | 8.95E-1 | 1.78E-2 | 5.59E+0 | 1.21E+0 | 0.16 |
| Benzene | 1.23E+0 | 4.47E-1 | 5.65E-1 | 3.73E+0 | 1.13E+0 | 0.14 |
| Methanol | 6.07E-1 | 3.16E-1 | 4.39E-3 | 2.39E+0 | 5.86E-1 | 0.15 |
| 2,2,4-Trimethylpentane | 4.77E-1 | 4.29E-1 | 2.87E-3 | 3.08E+0 | 3.91E-1 | 0.16 |
| 1,1,1-Trichloroethane (Methyl Chloroform) | 4.36E-1 | 1.85E-1 | 1.72E-1 | 1.31E+0 | 4.39E-1 | 0.16 |
| Hexane | 4.11E-1 | 3.06E-1 | 5.11E-3 | 2.97E+0 | 3.47E-1 | 0.16 |
| Methyl tert butyl ether | 3.04E-1 | 2.97E-1 | 6.46E-7 | 1.81E+0 | 2.49E-1 | 0.15 |
| Ethyl benzene | 2.95E-1 | 2.04E-1 | 4.15E-3 | 1.39E+0 | 2.53E-1 | 0.15 |
| Tetrachloroethylene (Perchloroethylene) | 2.30E-1 | 9.59E-2 | 6.54E-2 | 6.42E-1 | 2.38E-1 | 0.14 |
| Ethylene glycol | 2.14E-1 | 1.47E-1 | 1.49E-3 | 7.63E-1 | 1.91E-1 | 0.16 |
| Methyl isobutyl ketone | 1.94E-1 | 2.12E-1 | 2.36E-3 | 1.44E+0 | 1.29E-1 | 0.16 |
| 1,4-Dichlorobenzene (p-Dichlorobenzene) | 1.44E-1 | 5.10E-2 | 4.63E-2 | 3.41E-1 | 1.57E-1 | 0.15 |
| Chloroform | 1.29E-1 | 3.30E-2 | 5.96E-2 | 4.95E-1 | 1.29E-1 | 0.15 |
| 1,3-Butadiene | 1.10E-1 | 3.94E-2 | 3.97E-2 | 2.83E-1 | 1.09E-1 | 0.15 |
| Cyanide compounds | 8.41E-2 | 5.20E-2 | 6.52E-4 | 2.59E-1 | 7.64E-2 | 0.16 |
| Acrolein | 5.56E-2 | 2.60E-2 | 2.19E-3 | 1.62E-1 | 5.34E-2 | 0.16 |
| Chlorobenzene | 4.43E-2 | 3.32E-2 | 7.01E-6 | 2.08E-1 | 4.81E-2 | 0.15 |
| Propionaldehyde | 3.54E-2 | 2.15E-2 | 5.90E-4 | 1.46E-1 | 3.31E-2 | 0.16 |
| Trichloroethylene | 1.27E-2 | 1.21E-2 | 7.39E-3 | 2.67E-1 | 1.08E-2 | 0.06 |
| Ethyl chloride | 6.78E-3 | 4.32E-3 | 4.73E-5 | 2.53E-2 | 6.12E-3 | 0.16 |
| Cresols_Cresylic acid (isomers and mixture) | 4.28E-3 | 2.43E-3 | 9.18E-5 | 9.81E-3 | 3.84E-3 | 0.14 |
| Ethylene dichloride | 2.58E-3 | 5.59E-4 | 1.98E-3 | 8.93E-3 | 2.44E-3 | 0.13 |
| Cumene | 1.85E-3 | 1.21E-3 | 1.58E-5 | 6.30E-3 | 1.64E-3 | 0.16 |
| Triethylamine | 7.82E-4 | 5.58E-4 | 1.65E-6 | 3.42E-3 | 7.97E-4 | 0.16 |
| Propylene dichloride | 5.90E-4 | 1.39E-4 | 5.30E-4 | 3.83E-3 | 5.69E-4 | 0.03 |
| 4,4'-Methylene diphenyl diisocyanate (MDI) | 5.46E-4 | 3.99E-4 | 3.61E-6 | 1.90E-3 | 4.68E-4 | 0.16 |
| Vinyl acetate | 4.83E-4 | 3.67E-4 | 3.14E-6 | 4.96E-3 | 4.16E-4 | 0.15 |
| Isophorone | 3.65E-4 | 2.98E-4 | 0.00E+0 | 2.37E-3 | 3.66E-4 | 0.14 |
| Ethylene dibromide (Dibromomethane) | 1.65E-4 | 1.64E-5 | 1.34E-4 | 2.04E-4 | 1.73E-4 | 0.11 |
| Dibutylphthalate | 1.30E-4 | 1.00E-4 | 8.28E-7 | 6.29E-4 | 1.09E-4 | 0.16 |
| 2,4-Toluene diisocyanate | 8.17E-5 | 5.81E-5 | 5.57E-7 | 3.06E-4 | 7.06E-5 | 0.16 |
| Dimethyl phthalate | 3.88E-5 | 2.71E-5 | 2.75E-7 | 1.37E-4 | 3.43E-5 | 0.16 |
| *Heavy Metal(loid)s* |  |  |  |  |  |  |
| Lead compounds | 2.43E-3 | 1.90E-3 | 4.92E-4 | 3.45E-2 | 2.35E-3 | 0.09 |
| Manganese compounds | 1.33E-3 | 5.46E-4 | 5.81E-4 | 3.58E-3 | 1.32E-3 | 0.16 |
| Chromium Compounds | 7.43E-4 | 5.25E-4 | 4.34E-5 | 5.45E-3 | 7.16E-4 | 0.14 |
| Nickel compounds | 4.93E-4 | 4.13E-4 | 6.71E-5 | 4.18E-3 | 4.00E-4 | 0.12 |
| Arsenic compounds (inorganic, may include arsine) | 4.49E-4 | 2.77E-4 | 1.32E-4 | 1.84E-3 | 3.92E-4 | 0.16 |
| Cadmium compounds | 2.12E-4 | 1.87E-4 | 3.79E-5 | 1.20E-3 | 1.62E-4 | 0.15 |
| Mercury compounds | 2.03E-4 | 2.08E-4 | 1.02E-6 | 1.28E-3 | 1.47E-4 | 0.15 |
| Beryllium compounds | 1.87E-4 | 1.84E-4 | 1.86E-5 | 1.16E-3 | 1.38E-4 | 0.15 |
| *Other Pollutants* |  |  |  |  |  |  |
| Hydrochloric acid | 5.64E-2 | 4.55E-2 | 2.78E-4 | 5.25E-1 | 4.73E-2 | 0.06 |

**Table S2.** Bonferroni-corrected Pearson correlation coefficient of housing characteristics.

| *Variable* | Mobile Homes | Attached Homes | Home Gas Heating | Lacking Complete Plumbing | Built before 1940 |
| --- | --- | --- | --- | --- | --- |
| Attached Homes | -0.05 |  |  |  |  |
| Home Gas Heating | 0.14*** | -0.12* |  |  |  |
| Lacking Complete Plumbing | 0.16*** | 0.07 | 0.09 |  |  |
| Built before 1940 | 0.13** | 0.19*** | 0.19*** | 0.13*** |  |
| > 1 person per room | 0.25*** | 0.25 | -0.04 | 0.15*** | 0.29*** |

Note: **p*<0.05; ***p*<0.01; ****p*<0.001

**Table S3.** Negative binomial regression analyses of any and primary diagnosis of LRIs for ED visit and hospitalization rates and risk factors.

|  | Any Diagnosis LRIs | |  | Primary Diagnosis LRIs | | |
| --- | --- | --- | --- | --- | --- | --- |
|  | ED Visit | Hospitalization |  | ED Visit | Hospitalization |  |
| Risk Factors | IRR (95% CI) | IRR (95% CI) |  | IRR (95% CI) | IRR (95% CI) |  |
| *Simple Analyses* |  |  |  |  |  |  |
| Lower SES | 1.29 (1.25 - 1.34)*** | 1.47 (1.36 - 1.59)*** |  | 1.11 (1.09 - 1.13)*** | 1.44 (1.33 - 1.56)*** |  |
| Increased Air Pollution | 1.19 (1.15 - 1.24)*** | 1.13 (1.05 - 1.22)** |  | 1.03 (1.02 - 1.04)*** | 1.16 (1.08 - 1.25)*** |  |
| Mobile Homes (%) | 1.07 (1.05 - 1.09)*** | 1.05 (1.01 - 1.09)* |  | 1.05 (1.03 - 1.07)*** | 1.10 (1.06 - 1.15)*** |  |
| Attached Homes (%) | 0.99 (0.95 - 1.03) | 1.16 (1.08 - 1.26)*** |  | 1.07 (1.05 - 1.09)*** | 1.05 (1.01 - 1.10)** |  |
| Home Gas Heating (%) | 1.07 (1.04 - 1.11)*** | 1.20 (1.12 - 1.29)*** |  | 0.99 (0.96 - 1.03) | 1.14 (1.05 - 1.23)** |  |
| Lacking Complete Plumbing (%) | 1.09 (1.07 - 1.12)*** | 1.11 (1.06 - 1.17)*** |  | 1.07 (1.03 - 1.10)*** | 1.20 (1.12 - 1.29)*** |  |
| Built before 1940 (%) | 1.12 (1.10 - 1.15)*** | 1.24 (1.19 - 1.29)*** |  | 1.09 (1.06 - 1.11)*** | 1.11 (1.06 - 1.17)*** |  |
| > 1 person per room (%) | 0.91 (0.83 – 1.00)* | 0.92 (0.76 - 1.13) |  | 1.12 (1.10 - 1.14)*** | 1.22 (1.17 - 1.28)*** |  |
| Population Density (persons/sq. mile) | 1.11 (1.10 - 1.13)*** | 1.18 (1.14 - 1.22)*** |  | 1.05 (1.01 - 1.08)* | 0.80 (0.73 - 0.87)*** |  |
| In Pima County  *(Ref: Maricopa County)* | 1.04 (1.00 - 1.08)* | 0.76 (0.70 - 0.83)*** |  | 0.92 (0.85 - 1.01) | 0.80 (0.66 - 0.98)* |  |
|  |  |  |  |  |  |  |
| *Multiple Analysis* |  |  |  |  |  |  |
| Lower SES | 1.22 (1.15 - 1.30)*** | 1.51 (1.30 - 1.74)*** |  | 1.21 (1.13 - 1.28)*** | 1.52 (1.32 - 1.76)*** |  |
| Increased Air Pollution | 1.12 (1.06 - 1.19)*** | 0.91 (0.81 - 1.03) |  | 1.12 (1.06 - 1.19)*** | 0.90 (0.79 - 1.01) |  |
| Mobile Homes (%) | 1.03 (1.01 - 1.05)*** | 1.04 (1.01 - 1.08)* |  | 1.03 (1.01 - 1.05)** | 1.04 (1.01 - 1.08)* |  |
| Attached Homes (%) | 1.04 (1.02 - 1.06)*** | 1.03 (0.99 - 1.08) |  | 1.04 (1.02 - 1.06)*** | 1.04 (1.00 - 1.08) |  |
| Home Gas Heating (%) | 0.98 (0.95 - 1.02) | 1.11 (1.03 - 1.20)** |  | 0.98 (0.95 - 1.02) | 1.11 (1.03 - 1.20)** |  |
| Lacking Complete Plumbing (%) | 1.01 (0.98 - 1.04) | 1.08 (1.01 - 1.15)* |  | 1.01 (0.98 - 1.04) | 1.09 (1.02 - 1.16)* |  |
| Built before 1940 (%) | 1.02 (0.99 - 1.04) | 0.97 (0.92 - 1.02) |  | 1.02 (0.99 - 1.04) | 0.97 (0.92 - 1.02) |  |
| > 1 person per room (%) | 0.99 (0.96 - 1.02) | 1.07 (0.99 - 1.15) |  | 0.99 (0.96 - 1.02) | 1.05 (0.97 - 1.13) |  |
| Population Density (persons/sq. mile) | 0.93 (0.88 - 0.99)* | 0.49 (0.44 - 0.55)*** |  | 0.94 (0.89 – 1.00)* | 0.50 (0.45 - 0.57)*** |  |
| In Pima County  *(Ref: Maricopa County)* | 1.05 (0.93 - 1.18) | 0.54 (0.42 - 0.7)*** |  | 1.07 (0.95 - 1.21) | 0.48 (0.38 - 0.62)*** |  |

Note. CI = Confidence Interval; IRR = Incidence Rate Ratio; **p*<0.05; ***p*<0.01; ****p*<0.001
